# Gene Function Prediction from Functional Association Networks Using Kernel Partial Least Squares Regression

**DOI:** 10.1371/journal.pone.0134668

**Published:** 2015-08-19

**Authors:** Sonja Lehtinen, Jon Lees, Jürg Bähler, John Shawe-Taylor, Christine Orengo

**Affiliations:** 1 CoMPLEX, University College London, London, United Kingdom; 2 Institute of Structural and Molecular Biology, University College London, London, United Kingdom; 3 Department of Genetics, Evolution and Environment, University College London, London, United Kingdom; 4 Department of Computer Science, University College London, London, United Kingdom; Semmelweis University, HUNGARY

## Abstract

With the growing availability of large-scale biological datasets, automated methods of extracting functionally meaningful information from this data are becoming increasingly important. Data relating to functional association between genes or proteins, such as co-expression or functional association, is often represented in terms of gene or protein networks. Several methods of predicting gene function from these networks have been proposed. However, evaluating the relative performance of these algorithms may not be trivial: concerns have been raised over biases in different benchmarking methods and datasets, particularly relating to non-independence of functional association data and test data. In this paper we propose a new network-based gene function prediction algorithm using a **com**mute-time kernel and **pa**rtial least **s**quare**s** regression (Compass). We compare Compass to GeneMANIA, a leading network-based prediction algorithm, using a number of different benchmarks, and find that Compass outperforms GeneMANIA on these benchmarks. We also explicitly explore problems associated with the non-independence of functional association data and test data. We find that a benchmark based on the Gene Ontology database, which, directly or indirectly, incorporates information from other databases, may considerably overestimate the performance of algorithms exploiting functional association data for prediction.

## Introduction

### Network Approaches for Protein Function Prediction

The rapidly increasing volume of genomic and proteomic data has led to a surge of interest in the automatic extraction of functionally meaningful information from these datasets. One key approach is the *in silico* prediction of *gene and protein function*, a broad concept with meanings ranging from a protein’s biochemical role to its impact on phenotype. Owing to the scope of the problem, a variety of data sources and computational approaches have been exploited in gene function prediction. In general terms, prediction methods fall into two broad categories: *de novo* methods seeking to predict function based on intrinsic properties of a gene and *guilt-by-association* (GBA) approaches, which predict new functional labels based on a gene’s similarity to already functionally characterised genes.

A number of established GBA-type prediction methods base their predictions on sequence or structural similarity. Recently however, in response to the increasing prevalence of functional association data, there has been considerable interest in developing GBA methods exploiting functional association networks. The premise of these methods is that the functional similarity of two genes depends on 1) how close the genes are in the functional association network (local proximity) and 2) how many paths connect the two (global topology) [1]. There are two main classes of methods exploiting both global topology and local proximity: probabilistic network models and kernel methods.

Probabilistic network models are formalisms for representing dependencies between random variables. In the context of gene networks, these models capture how a gene’s function depends on that of its network neighbours. A number of approaches have modelled the problem in terms of belief propagation in these networks [2–5]. GeneMANIA [6], one of the most successful prediction algorithms to date [7, 8], makes use of this approach, implementing Gaussian label propagation. To our knowledge, no prediction algorithm has consistently outperformed GeneMANIA. We therefore benchmark our methods against this algorithm.

The other major class of methods makes use of kernels. Kernel approaches transform functional association networks into functional similarity scores between genes, based on the topology of the network. More specifically, these similarity scores represent inner products between gene vectors in some feature space, where distances between genes reflect their proximity in the network. A number of statistical learning approaches (such as various forms of regression for example) can be expressed in a form which operates on the kernel instead of the original feature space. Thus, kernel representations allow the use of statistical learning approaches on network data. Existing methods have most commonly used diffusion kernels, paired with support vector machines [9] or logistic regression [10]. A related method, FunctionalFlow [11], makes use of a diffusion kernel-like process.

While most existing methods have focused on diffusion kernels, recently, work by Heriche et al compared different kernel functions (i.e. different ways of generating similarity scores between genes from the network) [12]. In this work, the *commute time kernel* [13] was found to perform most robustly: when tested on a number of different benchmarks, this kernel was consistently among the top performers, while other kernels’ performance fluctuated significantly.

Heriche et al’s work made predictions by treating the kernel as a matrix of functional similarity scores between genes, but did not explore more complex prediction algorithms. Combining a commute time kernel with statistical learning methods therefore seems like a promising approach.

### Benchmarking

Accurate evaluation of the performance of prediction methods is essential for meaningful comparison of different algorithms. At a minimum, evaluation requires sets of true positives: genes which are known to be involved in the same function. These true positives are commonly derived from the Gene Ontology (GO) [14], with genes labelled with the same term considered a ‘set’ sharing the same function. A typical approach to benchmarking is to then use cross-validation: a subset of known labels are hidden, and the performance of the method is assessed by how well the hidden labels are recovered.

However, in the context of protein function prediction, cross-validation can be problematic. There is evidence to suggest that information is transferred between functional association databases (such as BioGrid [15], STRING [16] or KEGG [17] for example) and the GO: Gillis and Pavlidis looked at the source of GO annotations shared by proteins involved in a protein-protein interaction. 13% of these annotations were found to be derived from the publication which reported the interaction between the proteins [18]. Furthermore, the authors found a low (r = 0.2) but significant correlation between how well guilt-by-association methods perform for a particular term (as assessed by cross-validation) and the extent of this overlap between network and gene annotation data for this term. Thus, cross-validating a functional association network-based method may not actually reflect the algorithm’s ability to predict function for new genes, but rather the extent to which information has been dissipated across databases.

Interestingly, similar problems have also been reported for sequence similarity based prediction algorithms. The GO derives some of its annotations from sequence similarity (for example ‘IEA’ (inferred from electronic annotation), ‘ISS’ (inferred from sequence similarity)). Again, this raises the concern that the dataset used for evaluation is not independent from the dataset used for prediction, potentially leading to a biased estimation of predictive performance. Indeed, Rogers and Ben-Hur [19] showed that including these evidence codes when benchmarking a prediction algorithm tends to over-estimate how well sequence similarity based methods perform.

There have been significant efforts to compare prediction algorithms using a more realistic benchmark. Competitions such as CAFA (Critical Assessment of Function Annotation) [20] and MouseFunc [8] evaluate prediction methods based on novel true positives uncovered after the predictions have been made. Thus, unlike cross-validation, this benchmark directly assesses an algorithm’s ability to predict novel annotations.

These frameworks are essential for providing fair comparative assessment of prediction methods. However, CAFA-style competitions have also attracted criticism, particularly because of their reliance on GO annotations. There is evidence to suggest the process of label acquisition may be affected by existing annotations, which would extend the problems with cross-validation to benchmarks based on new labels as well. For example, existing ‘IEA’ annotations for a particular term are highly predictive of which genes will acquire an annotation with an experimental evidence code for the term [21], suggesting ‘IEA’ annotations may be guiding GO curation and/or target selection for experiments. This effect is strong: Gillis and Pavlidis predicted new labels based on existing ‘IEA’ annotations and reported performance comparable to the best CAFA entries in the 2011 competition [21]. This suggests that (sequence-based) computational methods may simply be re-creating the ‘IEA’ annotation and therefore seem to perform well, not because of actual predictive power, but because they mimic the process of annotations becoming incorporated in the GO.

We hypothesise that similar concerns may also be relevant for network-based prediction: the addition of new annotations into the GO may be affected by current functional association network data, either through temporal delays in information transfer into the GO or because choices of which putative gene/function pair to investigate may be partially driven by knowledge of functional associations.

### Our contribution

In this work, we develop a prediction algorithm based on a **com**mute-time kernel combined with a **pa**rtial least **s**quare**s** regression (Compass). PLS regression is a dimensionality reduction approach similar to principle component (PC) regression in that it projects the data into a subspace of the feature space. Instead of a space maximizing the variance of the inputs, however, PLS selects directions that maximize the covariance between inputs and the target. Originally developed for regression problems where features outnumber observations and exhibit multicollinearity PLS has been successfully applied to categorization problems [22], including ones involving genomic data [23, 24]. Indeed, given that high dimensionality and low sample size are common problems in the study of genomic and proteomic data [25], PLS is a promising approach for gene function prediction.

In addition to applying a novel approach to GBA prediction, we construct a simulation of a CAFA-style competition through a *rollback benchmark* [26]. We use functional association networks and GO assignments dating prior to a specific cut-off time to make predictions and evaluate these predictions on annotations acquired after the cut-off date. We use this benchmark to compare the Compass and GeneMANIA algorithms.

We also use the rollback benchmark to explicitly explore potential biases relating to transfer of information between databases. Furthermore, in light of the problems we identify, we develop two additional benchmarks (‘RNAi’ and ‘ageing’), which are not affected by information transfer. In these benchmarks, functionally related gene sets are derived from genes giving rise to a particular phenotype in a genome-wide knock-out experiment. The networks used in prediction pre-date the screens, ensuring information transfer between the test data and network is not possible. We use these benchmarks to further compare the performance of Compass and GeneMANIA.

## Methods

### Prediction

We implement a prediction algorithm (Compass) which first computes the **com**mute-time kernel of a functional association network and then performs a kernelized form of **pa**rtial least **s**quare**s** regression in the feature space represented by the kernel.

1. Networks are combined by summing the individual adjacency matrices: *A*(*i*, *j*) = ∑_*k*_ *A*_*k*_(*i*, *j*), where *A*_*k*_(*i*, *j*) is equal to the weight of the edge between nodes *i* and *j* in network *k*.
2. The commute-time kernel *K*_*CT*_ [13] of network with n nodes and adjacency matrix A is computed by: *K*_*CT*_ = *L* ^+^, where *L* ^+^ is the Moore-Penrose pseudoinverse of the graph laplacian *L*, defined by *L* = *D* − *A*, where *D* is the diagonal degree matrix, with entries $D(i,i)=\sum_{j=1}^{n}A(i,j)$. The commute-time kernel assumes the network has one connected component. In this work, if functional networks had more than one connected component, only the largest component was considered, as this resulted in the elimination of a small minority of the nodes. For networks with larger or more numerous smaller components, each component can be treated separately.
3. The kernel matrix is normalized:
  $$\begin{array}{c} K_{CT}^{norm}\left(i,j\right)=K_{CT}\left(i,j\right)/\sqrt{K_{CT}\left(i,i\right)*K_{CT}\left(j,j\right)} \end{array}$$
  and centred *K* ^*centred*^ = *K* − 1_*N*_ *K* − *K*1_*N*_ + 1_*N*_ *K*1_*N*_, where *K* is the normalized kernel and 1_*N*_ is a n-by-n matrix, where all elements are equal to 1/n.
4. For a gene set of interest (‘seed set’) for which we seek to predict new members, we generate a label vector *y*. If *n* is the set of all genes and *n*_+_ is the gene set of interest, $\hat{y}$ is constructed by assigning *y*(*i*) = 1, if *i* ∈ *n*_+_, else *y*(*i*) = 0 and then subtracting the mean, giving *y*(*i*) = 1 − ∣*n*_+_∣/∣*n*∣, if *i* ∈ *n*_+_, else *y*(*i*) = − ∣*n*_+_∣/∣*n*∣, where ∣*n*∣ is the total number of genes and ∣*n*_+_∣ the number of genes in the seed set. This approach treats all genes which are not part of the seed set as negative examples. Although more sophisticated methods of selecting negative examples exist, these tend to be GO specific. This did not suit our purpose of developing a method not restricted to GO label prediction. Note also that as PLS is multivariate, the approach could be extended to simultaneous prediction for multiple seed sets. This approach is not explored in this paper.
5. We perform a PLS regression in the feature space represented by the commute-time kernel using the kernelized implementation by Shawe-Taylor and Cristianini [27] The dependent variable predicted by the regression model, $\hat{y}$, gives the scores used to rank the genes for membership in the seed set.

The number of components to use in the PLS regression was determined by two-fold cross-validation on the seed set in the GO benchmark (i.e. based on labels discovered prior to the cut-off date). The optimal number of directions was 1 (see S1 Fig). This parametrization was used for all benchmarks.

### Network Construction

Functional association networks were downloaded from STRING database (version 8.1, released in June 2009) [16]. For each organism, this gave 7 individual networks, each corresponding to a different indicator of functional association (conserved genome neighborhood, gene fusion, phylogenic co-occurrence, co-expression, database imports, large-scale experiments and literature co-occurrence). STRING weights edges in the networks based on how well these interactions correspond to shared membership in KEGG pathways [17].

### Benchmarking

#### GO Rollback Benchmark

A GO rollback benchmark was constructed using data from yeast (*Saccharomyces cerevisiae*), using a 2009 cut-off date. Evaluation sets were created from the Biological Process (BP) branch of the GO tree. For each GO term, proteins for which the annotation was associated with a date prior to 2010 were taken as the seed set and those associated with a date from 2010 onwards as the test set. GO annotations were filtered by evidence code in order to 1) ensure high quality seed and test sets and 2) avoid predicted annotations, thus minimizing overlap between network data and test set. Specifically, only annotations derived from the evidence codes IC, IMP, TAS, IDA and NAS were used. Proteins not present in any of the functional association networks were ignored and categories with no proteins in the seed or novel set were excluded. This resulted 760 evaluation sets (i.e. GO terms).

#### RNAi Phenotypic Benchmark

For a complementary interpretation of function, we constructed a rollback phenotypic benchmark from genome-wide knock-out data by considering genes which, when knocked out, give rise to the same phenotype as a set of functionally related genes. Phenotypic data was downloaded from the GenomeRNAi database [28], a repository for RNAi screens. To ensure independence from the network data, only screens performed from 2010 onwards were considered. This benchmark was implemented in human and fly. Five fold cross validation was used to estimate predictive performance on this benchmark. Because of the independence of the network and test data, cross-validation on this benchmark is not subject to the concerns associated with cross-validation based on GO benchmarks.

#### Ageing Benchmark

Phenotypic benchmarking was also performed on an experimentally derived set of fission yeast (*Schizosaccharomyces pombe*) long-lived mutants from a longevity screen by Sideri et al [29] (see S1 Text for gene list). Predictions were seeded using long-lived mutant *clg1*, *pef1* [30], *pma1* [31], *sck2* and *pka1* [32], which were known prior to Sideri et al’s screen.

### Comparison to Genemania

GeneMANIA’s predictions were generated using the command line tool for the GeneMANIA cytoscape plug-in [33]. The plugin was given the same functional association networks and seed sets as used by our algorithm.

### Comparison to Multifunctionality and Degree-Based Prediction

Some authors have expressed concern that to some (potentially considerable) extent, the performance of guilt-by-association methods does not capture genuine *function-specific* insight, but instead reflects a general ranking of gene multifunctionality and/or degree [34]. Indeed, simply ranking genes based on their multifunctionality outperforms GeneMANIA on a disease gene prioritization task [34]. To investigate whether Compass outperforms this type of generalized ranking, we included a comparison against a degree-based and a multifunctionality-based ranking.

For the degree-based prediction, genes are ranked in order of their weighted node-degree in the combined String network. For the multifunctionality-based prediction, genes are ranked according to a multifunctionality score, defined, for gene *a*, as in the original publication [34] as:

$$\begin{array}{c} s_{a}=\sum_{i|a\in T_{i}}\frac{1}{|T_{i}\left|(n-\right|T_{i}\left|\right)} \end{array}$$

where *T*_*i*_ is GO term *i*, ∣*T*_*i*_∣ is the number of genes in GO term *i* and *n* is the total number of genes. This score is the number of GO terms a gene is labelled with, weighted by the contribution the gene makes to the group.

## Results and Discussion

### GO Rollback Benchmark

#### Relative performance of Compass and GeneMANIA

**Fig 1:**
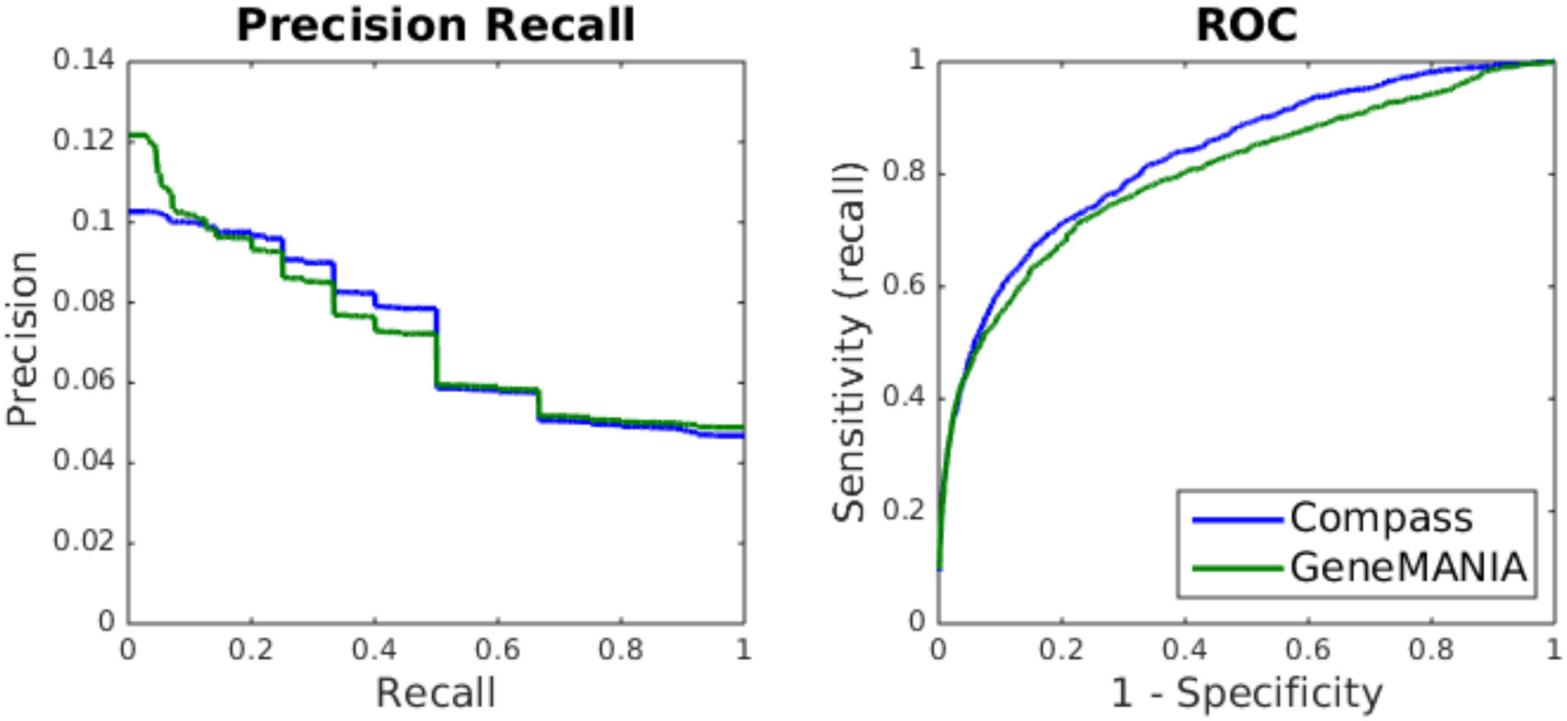
Average precision-recall and ROC curves for the GO rollback benchmark. The curves represent the average curve for the 760 GO terms.

The relative performance of Compass and GeneMANIA at predicting novel GO annotations was assessed using a rollback benchmark (Table 1 and Fig 1). Compass outperforms GeneMANIA when performance is measured in terms of AUC (*p* = 2.5 × 10^−4^, two-tailed Wilcoxon signed-rank test), while precision-based measures (mean average precision, *P*_*mean*_, and precision at recall 0.1, *P*_*r* = 0.1_) show no significant difference between the two algorithms (although both measures are higher for GeneMANIA). As shown in Fig 1, the average precision recall curves cross: on average, GeneMANIA performs better at low recall values while Compass performs better at high recall. This suggests Compass outperforming GeneMANIA on the AUC measure is associated with improved performance for gene-annotation pairings which are more difficult to predict.

**Table 1:** Predictive performance on the GO rollback benchmark.

Measure	Compass	GeneMANIA	p-value
AUC	0.8286 (sd 0.1861)	0.8026 (sd 0.2301)	2.5×10^−4^
*P*_*mean*_	0.0717 (sd 0.1838)	0.0718 (sd 0.1834)	0.0606
*P*_*r* = 0.1_	0.1000 (sd 0.2396)	0.1020 (sd 0.2462)	0.4725

Predictive performance of Compass and GeneMANIA, as measured by the area under the receiver operating characteristic (ROC) curve (AUC), mean average precision (*P*_*mean*_) and precision at recall 0.1 (*P*_*r* = 0.1_). The benchmark consists of 760 GO terms. Standard deviations (sd) are reported in parentheses.

To further understand the relative performance of Compass and GeneMANIA, we sought to identify factors affecting the performance of the algorithms. First, we looked at the size of the seed set (i.e. number of training examples) and the specificity of the GO term (in terms of GO level). There was no significant correlation between the number of seed genes and performance for either method. For both methods, performance correlated with GO specificity, with higher performance at greater specificity (Spearman correlation coefficient 0.1740 and 0.1459 for Compass and GeneMANIA respectively *p* < 10^−4^). However, as this effect is similar for both methods, it is unlikely to explain the difference in performance.

**Fig 2:**
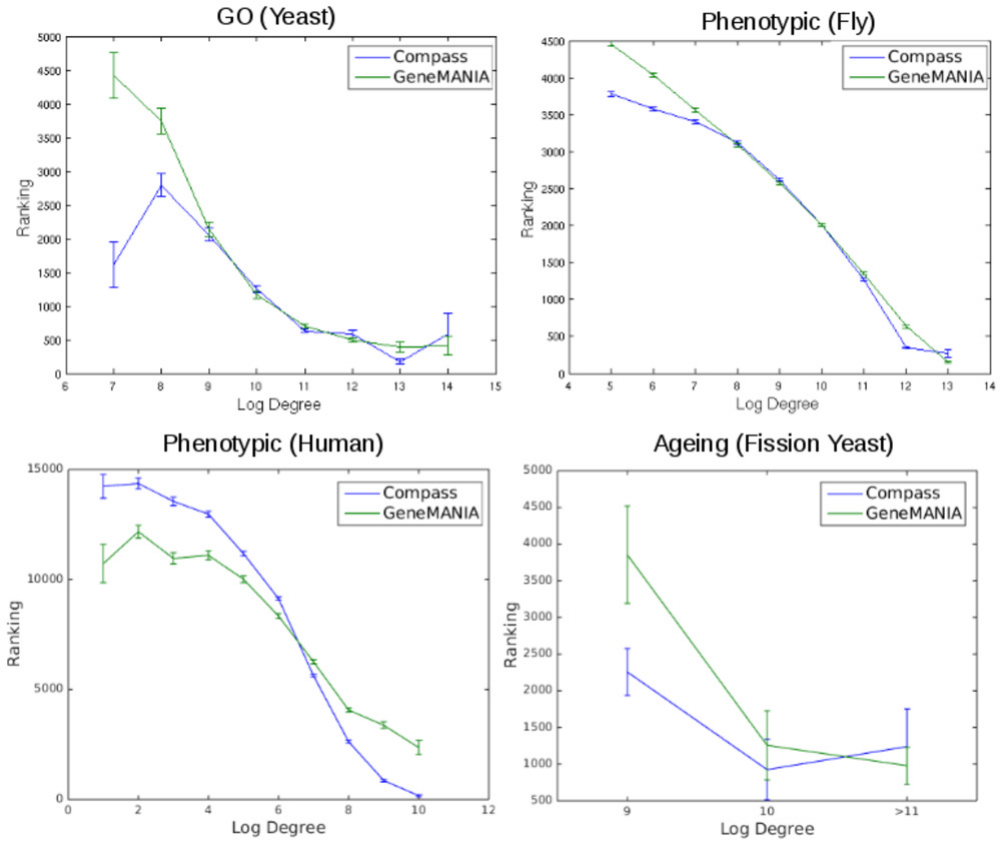
Association between a gene’s degree and how well the algorithms predict new annotations for the gene. The figure shows average ranking (top ranking = rank of 1.) for genes grouped according to weighted degree in the String network for all four benchmarks (GO Benchmark, RNAi benchmark in fly and human and fission yeast ageing benchmark). Error bars represent standard error of the mean.

Second, we looked at how a gene’s degree affects how successful the algorithms are at predicting annotations for it. As shown in Fig 2, both methods are, as expected, more successful at making predictions for high degree genes. However, Compass outperforms GeneMANIA for genes with very low degree. This suggests that Compass’ improved performance on difficult to predict gene-annotation pairings arises from its improved performance on low degree genes. Compass outperforms GeneMANIA for low-degree genes in three out of four of our benchmark sets (Fig 2). It is thus unclear whether this is a general property of the method or a particularity of the benchmark sets used.

#### Comparison to Degree and Multifunctionality-Based Rankings

Network-based predictors are known to be biased toward high degree genes (Fig 2). This has led to a concern that instead of capturing genuine functional insight, network-based methods may simply be producing a *generic* ranking based on node-degree and/or gene multifunctionality [34]. Indeed, simply ranking genes by multifunctionality (based on the number of GO terms each gene is labelled with) has been reported to outperform GeneMANIA on a disease gene prioritization task [34]. We therefore tested how degree and multifunctionality-based rankings perform on this benchmark. Both generic rankings are clearly outperformed by Compass and GeneMANIA (AUC 0.5940 and 0.5719 for degree and multifunctionality-based rankings respectively). Thus, while a generic ranking gives above random performance (reflecting the tendency of central and well annotated genes to acquire new rankings), the guilt-by-association methods do provide further, function-specific insight on this benchmark.

#### GeneMANIA Weighting Scheme

The default option for the GeneMANIA algorithm is to integrate multiple networks using query-specific weights, which reflect how well a network captures functional similarity between the query genes. For Compass, on the other hand, networks were combined without query-specific weighting. To ensure that the observed difference in performance was not simply due to GeneMANIA’s query-specific weighting of the networks, GeneMANIA was also run without the seed-specific weighting step. The relative performance of the two algorithms was not affected: removal of seed-specific weighting decreased GeneMANIA’s performance to AUC 0.800 (compared to 0.803 with the default setting and 0.8286 for Compass).

#### Cross-Validation vs Rollback with GO Benchmark

**Fig 3:**
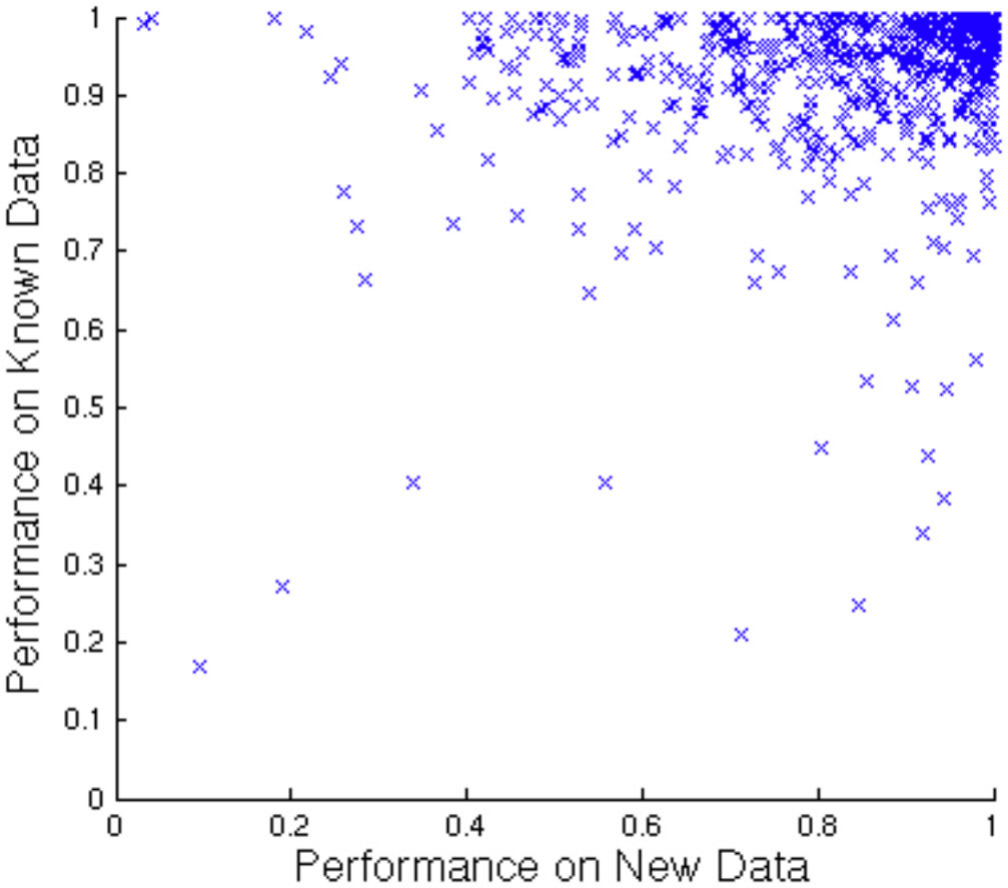
Performance on known vs new labels. Comparison between Compass performance on new data and known data (as measured by two-fold cross validation) on the GO benchmark. Each data point represents performance on one GO term as measured by AUC.

Constructing the rollback benchmark gave us the opportunity to explicitly explore potential biases in how GO-based benchmarks assess predictive performance. We started by comparing the rollback to cross-validation. As discussed in the introduction, there are concerns that cross-validation using known labels does not adequately capture how well GBA methods predict *new* annotations. To assess this, we compared how Compass performed on the rollback benchmark with its performance as evaluated by cross-validation using the labels acquired prior to the cut-off date. As expected, performance was higher using cross-validation (see Fig 3), suggesting information transfer between functional association networks and the known labels makes the known labels ‘too easy’ to predict during cross-validation.

Furthermore, the correlation between AUC (area under receiver operating characteristic curve) as evaluated by cross-validation and using the rollback benchmark was relatively low (Pearson’s correlation coefficient of 0.260). This indicates that cross-validation on known protein sets is not a particularly good indicator of performance at predicting novel labels.

#### Effect of Discovery Date on Label Predictability

**Fig 4:**
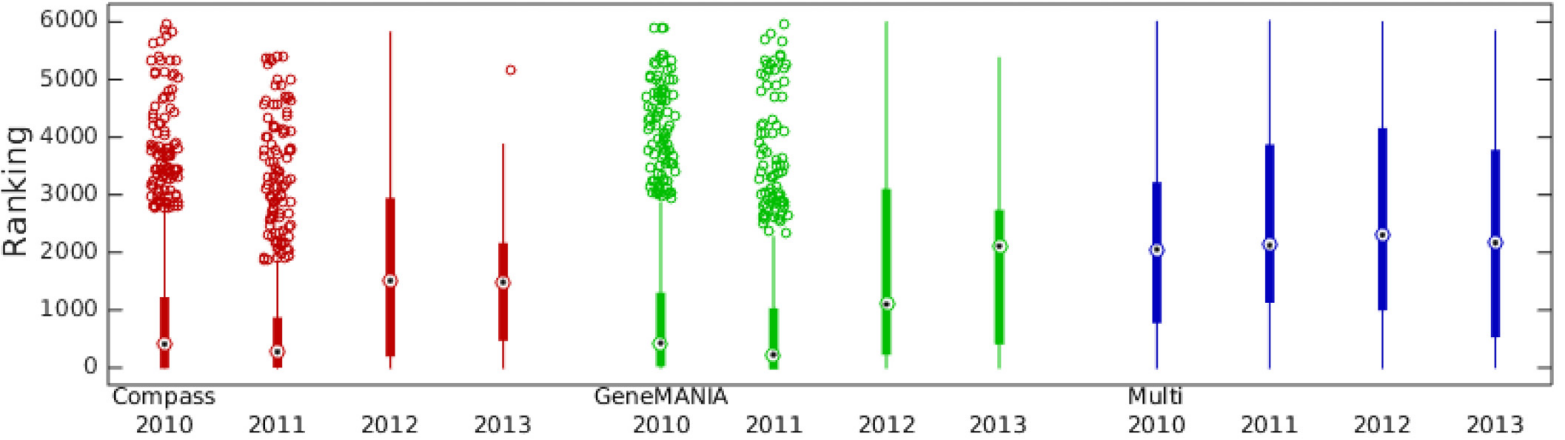
Relationship between how easy an annotation is to predict and the year the annotation was made in the GO. The ease of prediction is measured as the ranking of the gene in a prioritized list (i.e. a rank of 1 indicates the highest prioritized gene). The relationship is shown for Compass (red), GeneMANIA (green) and a multifunctionality-based predictor (blue).

As discussed in the introduction, a rollback benchmark does not necessarily guarantee independence between the network and the test data. If currently known functional associations do indeed drive label acquisition, we would expect the date a new label was acquired to affect how easily it is retrieved: labels acquired close to the cut-off date would be easier to predict than those acquired later. We therefore looked at the correlation between how highly true positives were ranked and the date the annotation was made (see Fig 4).

There is a significant positive correlation between the ranking and the annotation date for both Compass and GeneMANIA (Spearman Correlation Coefficient (SCC) 0.197 and 0.163 respectively, *p* < 10^−15^), indicating early new labels are indeed easier to predict (top ranking = rank of 1, see Fig 4).

#### Temporal Effects by Information Source

The STRING network is composed of functional associations from a number of sources: genome neighbourhood, gene fusion, genome co-occurrence, co-expression, experiments (i.e. high-throughput screens for physical interaction), databases (i.e. curated small-scale interaction screens and annotated pathways) and textmining. Some of these evidence types, such as textmining, may be more susceptible to the temporal effects described above. We therefore looked at the correlation between label discovery date and predictability in each source network individually (Table 2).

**Table 2:** Correlation between label predictability and date of discovery in different network types.

	C	C	G	G
	SCC	p	SCC	p
Neighourbood	0.03	5.2×10^−1^	-0.11	4.5×10^−2^
Gene Fusion	-0.19	2.2×10^−2^	-0.20	1.8×10^−2^
Cooccurrence	-0.26	6.5×10^−3^	-0.13	1.6×10^−1^
Coexpression	-0.07	2.3×10^−2^	0.06	6.1×10^−2^
Experiments	**0.15**	1.3×10^−15^	**0.15**	2.0×10^−15^
Databases	**0.14**	1.2×10^−5^	**0.20**	3.8×10^−10^
Textmining	**0.13**	1.2×10^−9^	**0.08**	3.0×10^−4^

Spearman correlation (SCC) between the ranking of true positive annotations and the date the annotation was made in the GO rollback benchmarks for Compass (C) and GeneMANIA (G). A positive correlation indicates early labels are easy to predict because a low numerical rank (example rank = 1) indicates an easily predictable gene. Significant correlations (*p* < 0.0037, derived using a Sidak multiple comparison correction $\alpha =1-{\left(1-\bar{\alpha }\right)}^{1/k}$, where *k* is the number of comparisons (14) and *α* the original significance level (0.05)) have been highlighted (bold).

After correction for multiple testing using Sidak correction (the corrected significance level α = 0.0037, given by $\alpha =1-{\left(1-\bar{\alpha }\right)}^{1/k}$, where *k* is the number of comparisons (14) and $\bar{\alpha }$ is the original significance level (0.05)), the experiment, database and textmining networks all showed significant positive correlations between ranking and discovery date (i.e. earlier labels were easier to predict).

It is not surprising that the effect is seen in these three networks, because these networks represent known protein interactions, whereas genome neighbourhood, gene fusion and co-occurrence networks are classed as *de novo* interaction prediction methods [16]. Indeed, if the temporal effect is due to a lag in information transfer between databases, we would not expect to observe a correlation between discovery date and ease of prediction for the *de novo* networks: GO annotations inferred from these functional associations would be classed as non-experimental and would therefore be excluded from our rollback benchmark which only included experimental annotations. If, on the other hand, the temporal effect is due to the publicly available functional association data guiding choices of targets for experiments, we would also expect the effect to be clearest in the experimental, database and textmining networks as these information sources are more widely used than the other four.

### Phenotype-Based Independent Benchmarks

The results from the GO rollback benchmark are relevant for the design of CAFA or MouseFunc style competitions because they suggest the time period between prediction and assessment (i.e. the time window allowed for new annotations to accumulate) could affect how well a method appears to perform. This issue is particularly noteworthy because genes not labelled with a particular term at the time of evaluation are considered negatives although they could actually represent hidden true positives [35]. This could lead to penalisation of methods ranking the ‘more difficult’ and not yet discovered labels higher than the more obvious ones. This leads to the concern that competition style benchmarks may encourage the building of tools to mimic experimental discovery as opposed to guiding it. Thus, the re-evaluation of algorithms after a longer wait period could provide valuable insight into their performance. Indeed, CAFA has been designed to allow reassessment of algorithm performance at a later date [20].

As an alternative way of addressing the concerns discussed above, we designed two benchmarks where the network data and the test data were definitely independent. As before, we used the 2009 STRING functional association networks. The gene sets used for testing were derived from genome-wide knock-out screens performed *after this date*. Test sets consisted of genes giving rise to a particular phenotype when knocked-out. This principle was used to construct two benchmarks: one based on RNAi screens in fly and human and one based on a screen for ageing related genes in fission yeast.

#### RNAi Benchmark

**Fig 5:**
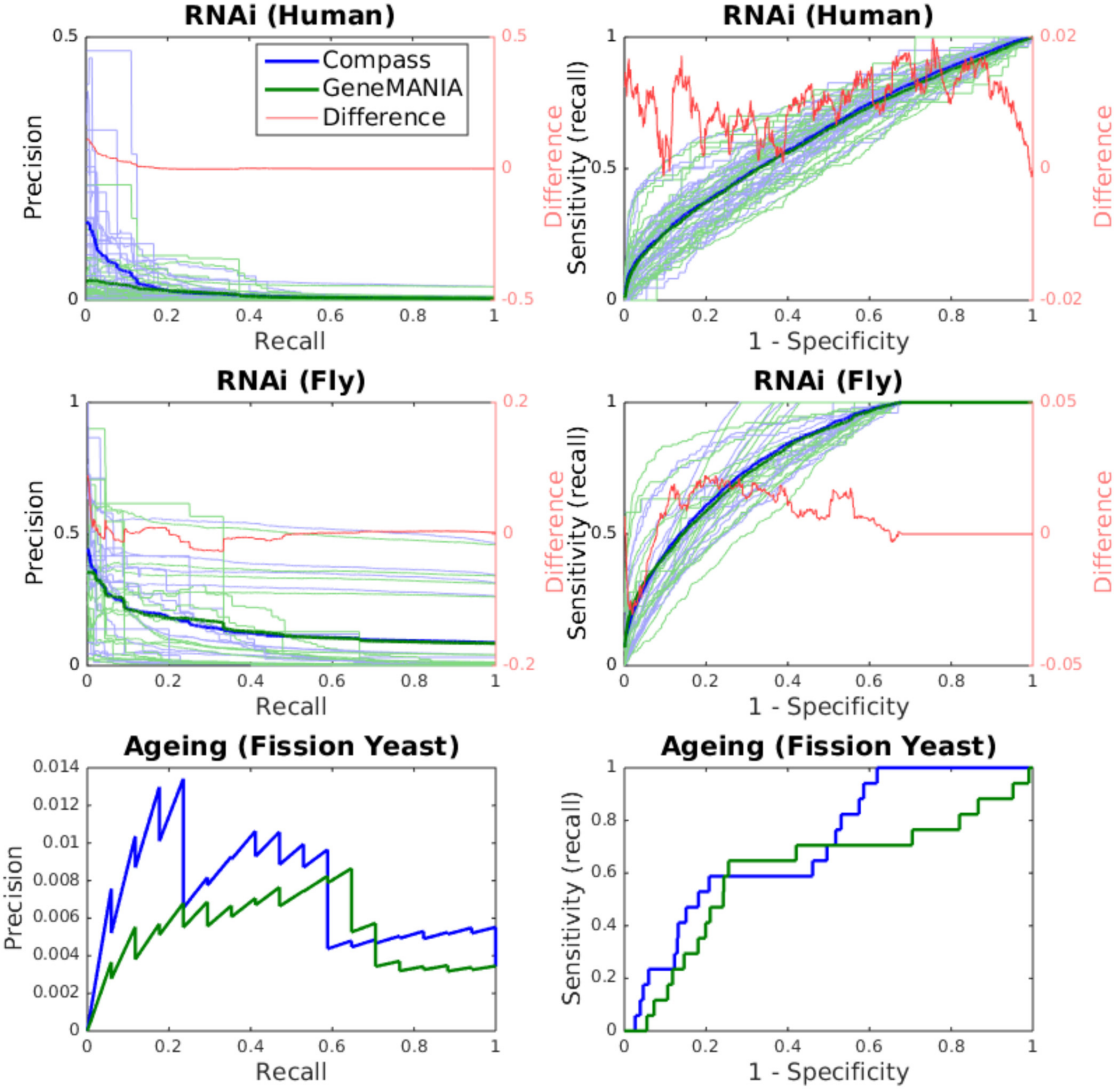
Precision-recall and ROC curves for the phenotypic benchmarks (RNAi and Ageing) for Compass and GeneMANIA. Precision-recall (right) and ROC (left) are shown for all gene sets (27 for human and 18 for fly) in the RNAi benchmark (top two rows). Average curves are also shown, as well as the average difference between the two methods (Compass minus GeneMANIA). The bottom row shows precision-recall and ROC curves for the fission yeast ageing benchmark (one gene set). The precision-recall curves for the plots depicting multiple gene sets (top two rows) have been interpolated for clarity.

This benchmark was based on a database of phenotypes observed in various RNAi knock-out screens. As shown in Fig 5 and summarised in Table 3, Compass significantly outperforms GeneMANIA on this benchmark.

**Table 3:** Summary of Compass and GeneMANIA performance on the phenotype-based benchmarks.

Benchmark/Measure	COMPASS	GeneMANIA	p-value
AUC RNAi (Human and Fly)	0.6542 (sd 0.0773)	0.6442 (sd 0.0927)	0.0175
*P*_*mean*_RNAi (Human and Fly)	0.0628 (sd 0.1101)	0.0593 (sd 0.1103)	0.0051
*P*_*R* = 0.1_ RNAi (Human and Fly)	0.1127 (sd 0.1725)	0.0965 (sd 0.1568)	0.0019
AUC Ageing (Fission Yeast)	0.713	0.613	N/A
*P*_*mean*_Ageing (Fission Yeast)	0.0082	0.0057	N/A
*P*_*R* = 0.1_ Ageing (Fission Yeast)	0.0104	0.0055	N/A

Predictive performance of Compass and GeneMANIA, as measured by the area under the receiver operating characteristic (ROC) curve (AUC), mean average precision (*P*_*mean*_) and precision at recall 0.1 (*P*_*r* = 0.1_), on the phenotype-based benchmarks. The RNAi benchmark consists of 45 gene sets (27 for human and 18 for fly). Standard deviations (sd) are reported in parenthesis. The ageing benchmark consists of 17 genes associated with a long lived phenotype in a knock-out screen. For the RNAi benchmark, p-values are derived from a two-tailed Wilcoxon ranked sum test. For the ageing benchmark (which consists of only one gene set), the statistical significance of the difference in performance was evaluated by comparing how highly each long lived mutant was ranked by Compass and Genemania, giving a p-value of 0.0168 (two-tailed Wilcoxon sing-rank test).

#### Ageing Benchmark

This benchmark was based on long lived mutants (n = 17) identified in a genome wide screen [29]. Prediction was seeded with long lived mutants known prior to the screen (see Methods). On this benchmark, COMPASS outperforms GeneMANIA (Fig 5 and Table 3). The statistical significance of this result was evaluated by comparing how highly each long lived mutant was ranked by Compass and Genemania, giving a p-value of 0.0168 (two-tailed Wilcoxon sing-rank test).

### Conclusion

We have proposed a novel guilt-by-association prediction algorithm (Compass) for gene function prediction which outperforms GeneMANIA, a leading network-based prediction algorithm, on a CAFA-style GO-based benchmark and two phenotype-based benchmarks.

Additionally, we have shown that information transfer between databases may affect not only cross-validation, but also benchmarks based on the accumulation of new labels, such as CAFA-style competitions. Our results suggest the length of the wait period between prediction and evaluation affects how well algorithms appear to perform. Thus, re-evaluation of prediction methods after a longer wait period may provide further insight into the relative performance of algorithms. We have also proposed an alternative, phenotype-based, benchmark which may, in context where knock-out phenotype is a relevant indicator of protein function, serve as a complementary assessment method.

## Supporting Information

S1 Fig Compass parametrization. Compass performance on the GO benchmark set, using different number of dimensions for the PLS regression. Performance is measured by area under ROC curve (AUC). Performance is shown estimated from cross-validation on the seed set (‘seed’) and prediction of new labels (‘new’). Error bars represent standard error of the mean. The number of optimal dimensions between seed set and novel set is the same: performance is maximized using a single dimension. This is in line with previous work recommending the use of K-1 dimensions for PLS discriminant analysis, where K is the number of classes [24].
(TIF)

S1 Text Gene list for ageing benchmark. Experimentally derived set of fission yeast (*Schizosaccharomyces pombe*) long-lived mutants from a longevity screen by Sideri et al [29].
(TXT)
