# Supplementary figures and images for: Gene Function Prediction from Functional Association Networks Using Kernel Partial Least Squares Regression
Source: PLoS One. 2015 Aug 19;10(8):e0134668. doi: 10.1371/journal.pone.0134668 (PMC4545790; doi:10.1371/journal.pone.0134668)

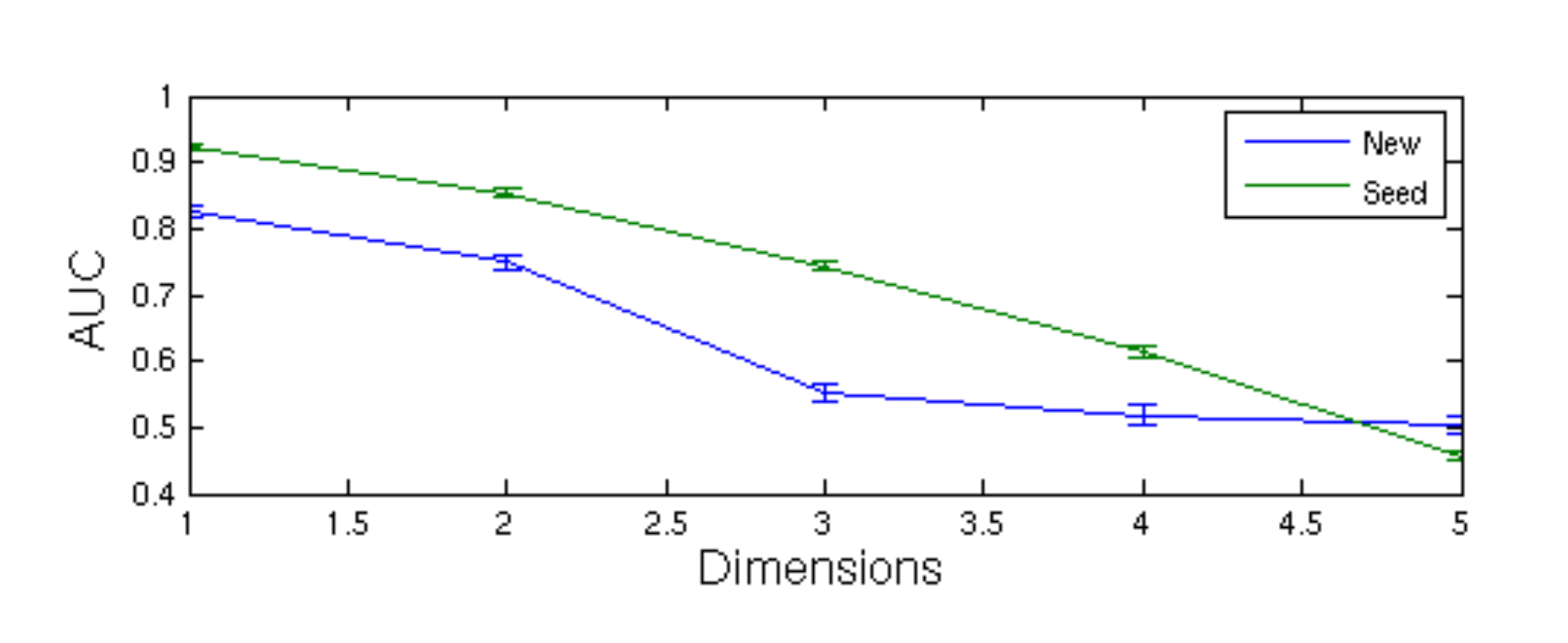

Supplement: S1 Fig — Compass performance on the GO benchmark set, using different number of dimensions for the PLS regression. Performance is measured by area under ROC curve (AUC). Performance is shown estimated from cross-validation on the seed set (‘seed’) and prediction of new labels (‘new’). Error bars represent standard error of the mean. The number of optimal dimensions between seed set and novel set is the same: performance is maximized using a single dimension. This is in line with previous work recommending the use of K-1 dimensions for PLS discriminant analysis, where K is the number of classes [24]. (TIF) [file pone.0134668.s001.tif]
